# A Remedy for Every Ill

**Published:** 1876-08

**Authors:** 


### *A REMEDY FOR EVERY ILL.*

It is a popular idea that there is, in the vegetable kingdom, a specific for each and every bodily infirmity, and that the time will come when all diseases will be cured by their appropriate remedies.

But the investigations of this age of reason have spoiled many attractive theories, and this among the rest. The most careful experiments have proved beyond a doubt that of all the so-called remedies described in the "dispensatory," not one in a hundred possesses any medicinal property; and of those that do, we can count on our fingers the number that are of any value in the treatment of diseases. Let us suppose, however, for the sake of the argument, that all these plants are medicinal; still the idea that the Creator has provided a cure for every ill seems absurd when we consider that most of the ailments described by physicians are not *diseases*, but only disturbances that indicate disease in some vital organ. Here is the dividing line between medical skill and presumptuous quackery. The educated physician locates the disease from the symptoms, and the quack mistakes the symptoms for the disease. Still the quack has the majority with him even in this Centennial year, and in the last years of the nineteenth century. There are at least two causes for this. The majority won't reason. Then, when a fool proclaims himself a wise man, half the world believes it, and the other half won't take the trouble to dispute it.

